# Production of PHB From CO_2_-Derived Acetate With Minimal Processing Assessed for Space Biomanufacturing

**DOI:** 10.3389/fmicb.2021.700010

**Published:** 2021-07-28

**Authors:** Stefano Cestellos-Blanco, Skyler Friedline, Kyle B. Sander, Anthony J. Abel, Ji Min Kim, Douglas S. Clark, Adam P. Arkin, Peidong Yang

**Affiliations:** ^1^Center for the Utilization of Biological Engineering in Space, Berkeley, CA, United States; ^2^Department of Materials Science and Engineering, University of California, Berkeley, Berkeley, CA, United States; ^3^Department of Bioengineering, University of California, Berkeley, Berkeley, CA, United States; ^4^Department of Chemical and Biomolecular Engineering, University of California, Berkeley, Berkeley, CA, United States; ^5^Lawrence Berkeley National Laboratory, Molecular Biophysics and Integrated Bioimaging Division, Berkeley, CA, United States; ^6^Lawrence Berkeley National Laboratory, Environmental Genomics and Systems Biology Division, Berkeley, CA, United States; ^7^Department of Chemistry, University of California, Berkeley, Berkeley, CA, United States; ^8^Lawrence Berkeley National Laboratory, Chemical Sciences Division, Berkeley, CA, United States; ^9^Kavli Energy NanoSciences Institute, University of California, Berkeley, Berkeley, CA, United States

**Keywords:** biomanufacturing, CO_2_ reduction, biopolymer, acetogen biocatalyst, *in situ* resource utilization

## Abstract

Providing life-support materials to crewed space exploration missions is pivotal for mission success. However, as missions become more distant and extensive, obtaining these materials from *in situ* resource utilization is paramount. The combination of microorganisms with electrochemical technologies offers a platform for the production of critical chemicals and materials from CO_2_ and H_2_O, two compounds accessible on a target destination like Mars. One such potential commodity is poly(3-hydroxybutyrate) (PHB), a common biopolyester targeted for additive manufacturing of durable goods. Here, we present an integrated two-module process for the production of PHB from CO_2_. An autotrophic *Sporomusa ovata (S. ovata)* process converts CO_2_ to acetate which is then directly used as the primary carbon source for aerobic PHB production by *Cupriavidus basilensis (C. basilensis)*. The *S. ovata* uses H_2_ as a reducing equivalent to be generated through electrocatalytic solar-driven H_2_O reduction. Conserving and recycling media components is critical, therefore we have designed and optimized our process to require no purification or filtering of the cell culture media between microbial production steps which could result in up to 98% weight savings. By inspecting cell population dynamics during culturing we determined that *C. basilensis* suitably proliferates in the presence of inactive *S. ovata*. During the bioprocess 10.4 mmol acetate L ^–1^ day^–1^ were generated from CO_2_ by *S. ovata* in the optimized media. Subsequently, 12.54 mg PHB L^–1^ hour^–1^ were produced by *C. basilensis* in the unprocessed media with an overall carbon yield of 11.06% from acetate. In order to illustrate a pathway to increase overall productivity and enable scaling of our bench-top process, we developed a model indicating key process parameters to optimize.

## Introduction

Space exploration remains a key aspect of technical and scientific programs of multiple nations (ISECG, 2018). For instance, the National Aeronautics and Space Administration (NASA) has received increased federal funding in the United States as strategic investments call for expansion of crewed space exploration capabilities (National Research Council, 2012, 2014). These efforts are highlighted by the newly established Artemis Program which aims to land women and men on the Moon and Mars, as well as various directorates to fund research into systems enabling human-led deep space exploration (Northon, 2017; NASA, 2018, 2019).

However, the exorbitant costs to transport goods into space represent a major roadblock for space exploration (Wertz and Larson, 1996; Linck et al., 2019). Notably, as human-based mission lengths increase so does the demand for consumables (Moore, 2010). Additionally, tenuous re-supply lines to faraway locations like Mars could be easily disrupted (Tanner et al., 2006). Therefore, it is pivotal to transition from missions that solely rely on re-supplied or pre-deployed stores of single-use consumables to those that sustainably produce and recycle consumables.

*In situ* resource utilization (ISRU) is the practice to generate products in space from local materials and chemicals. The purpose of ISRU is mainly to support astronauts on long expeditions and could provide products and materials for life support, construction, propellants, and energy harvesting (Linne et al., 2017). This would also allow for the transport of more goods that would be prohibitively difficult to generate in space (e.g., photovoltaics and experimental equipment) and allow for production flexibility given a change of mission demand. Successful implementation of ISRU will require the harmonious integration of new technological platforms interfacing biotechnology, systems engineering, solar energy harvesting, agriculture, remediation, and manufacturing (Berliner et al., 2020).

There has been heightened interest and research into microorganisms as the core platform to generate consumables and life-support materials in space (Menezes, 2018; Nangle et al., 2020). Autotrophic and diazotrophic microorganisms fix CO_2_ and N_2_, both of which are present in the Martian atmosphere (Owen et al., 1977; Ragsdale and Pierce, 2008). Single-celled organisms replicate and self-repair; thus, only dried inocula would need to be transported. Bacteria have been reported to produce value-added products including bioplastics, biofuels and pharmaceuticals from CO_2_, fix N_2_ to fertilizer and aid in waste remediation (Kobayashi and Kobayashi, 2006; Liu et al., 2015, 2016, 2017; Soundararajan et al., 2019; Su et al., 2020). Many strains may be powered directly by solar-generated electricity or by electrochemically produced reducing equivalents such as H_2_ (Sakimoto et al., 2018; Cestellos-Blanco et al., 2020). Here, we present a bacteria-based bioprocess to renewably produce a biopolymer poly(3-hydroxybutyrate) (PHB) from CO_2_ while optimizing for ISRU specific requirements.

Poly(3-hydroxybutyrate) is a type of polyhydroxyalkanoate (PHA) belonging to a class of bio-polyesters (Sudesh et al., 2000). It accumulates in different bacterial cells and archaea intracellularly triggered by physiological stress when lacking certain nutrients (Koller, 2019; Müller-Santos et al., 2020). Bacteria employ the biopolymer resulting from carbon assimilation of glucose, starch and organic acids as a form of carbon and energy storage as well as a protectant against stressors (Sheu et al., 2009; Obruca et al., 2018; Dalsasso et al., 2019). PHB is synthesized by the reduction of two condensed acetyl-CoA molecules to produce the hydroxybutyryl-CoA monomer. PHB is biodegradable and has material properties resembling those of polyethylene (McAdam et al., 2020; Meereboer et al., 2020). Additive manufacturing techniques such as 3D-printing can employ PHB that has been extracted from cells and pelletized (Alarfaj et al., 2015; Vigil Fuentes et al., 2020; Kovalcik, 2021). Altogether, PHB represents a viable pathway for biomanufacturing of material products in space.

Acetogenic bacteria fix CO_2_ to acetate and biomass through anaerobic respiration. The Wood–Ljungdhal pathway in acetogens undertakes the reduction of CO_2_ to acetyl-CoA which is then used in biosynthesis or converted to acetate gaining ATP. Other minor products of the pathway may include ethanol, 2,3-butanediol, and hexanoic acid depending on growth conditions (Fernández-Naveira et al., 2017). Acetogens obtain electrons directly from the reduction of minerals, or through the oxidation of electron shuttles or reducing equivalents. Certain acetogens like *Sporomusa ovata* (*S. ovata*) have been found to directly obtain electrons from a poised cathode or from electrochemically generated reducing equivalents like H_2_ (Nevin et al., 2011; Rodrigues et al., 2019). This enables CO_2_ bioelectrosynthesis to acetate with high selectivity and long-term stability.

**FIGURE 1:**
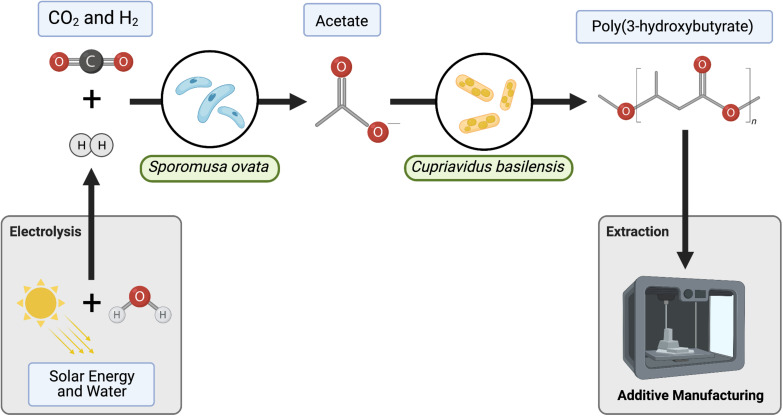
Process diagram for two-stage bioprocess with media recycle. *Sporomusa ovata* oxidizes H_2_ to fix CO_2_ into acetate. *C. basilensis* grown in the unprocessed spent medium converts acetate to Poly(3-hydroxybutyrate) (PHB). Modules to be tested for end-to-end biomanufacturing including H_2_O reduction and PHB extraction to 3D printing in gray.

We have employed *S. ovata* as a self-replicating and self-regenerating biocatalyst to fix CO_2_ to acetate. We then selected *Cupriavidus basilensis* (*C. basilensis*) for its ability to consume acetate as a primary carbon feedstock (Figure 1; Wierckx et al., 2010; Friman et al., 2013). In addition, *C. basilensis* produces PHB with high carbon efficiency, and as a significant proportion of biomass. Few accounts have been reported of PHB/PHA production starting from CO_2_ as a carbon source (Liu et al., 2015; Pepè Sciarria et al., 2018). And several components of these accounts do not translate well to ISRU-based space production including the low rate of CO_2_ fixation and the requirement to purify and concentrate the intermediate carbon molecules before their use in PHA bioproduction. *S. ovata* can directly take up electrons from a cathode for CO_2_ to acetate conversion but this approach is limited by the alkaline pH change at the cathode and the bacteria/electrode interface (Lovley and Nevin, 2013). Therefore, we employ H_2_ generated efficiently in a separate module to circumvent these challenges. Direct CO_2_ bioelectrosynthesis to PHB has also been reported (Liu et al., 2016). However, the segmented approach (CO_2_ to acetate, acetate to PHB) prevents incompatibilities that arise from combining electrochemistry and biological catalysts such as the requirement to maintain neutral pH. Additionally, a segmented process flow allows for individual unit optimization and overall biomanufacturing modularity (Fast and Papoutsakis, 2012; Kiefer et al., 2021). For these reasons, we have designed our bioprocess to include a base medium used for both bioreactions–acetate and PHB generation. The ability to directly recycle media circumvents the need for acetate purification and concentration steps which require significant non-ISRU resources (Supplementary Table 4). Altogether, we demonstrate peak acetate production from CO_2_ of 10.4 mmol acetate L ^–1^ day^–1^ which translates to a titer of acetate of 25 mM in 2.5 days. This is sufficient for the generation of 12.54 mg PHB L^–1^ hour^–1^. Through modeling this process, we have determined that we can reduce the time to generate 25 mM of acetate by 75%. This can be accomplished by increasing the gas to liquid mass transfer of H_2_ through improvements in bioreactor design.

## Materials and Methods

### Cultivation of *Sporomusa ovata*

*Sporomusa ovata* was obtained from the American Type Culture Collection (ATCC 35899) and rehydrated as indicated. It was then grown in DSMZ 311 medium and aliquoted with DMSO as a cryoprotectant at −80°C. Frozen cells were inoculated in DSMZ 311 medium and cultured for two cycles before inoculating in yeast medium (DSMZ 311 medium, casitone, betaine, and resazurin omitted). *S. ovata* from yeast medium were inoculated at 5% (v/v) in autotrophic *S. ovata* medium (DSMZ 311 medium, casitone, betaine, resazurin, and yeast omitted) with H_2_/CO_2_ 80/20% headspace at 30 PSI. *S. ovata* was consistently incubated at 35 °C in starting pH 7.2 and an orbital shaker was employed for autotrophic growths. Acetate concentration was monitored by 1H-qNMR spectroscopy with sodium 3- (trimethylsilyl)-2,2′,3,3′-tetradeuteropropionate as the internal standard. *S. ovata* from yeast cultures were also inoculated in deoxygenated DM9 (Supplementary Table 1) at 10% (v/v) with H_2_/CO_2_ 80/20% headspace at 30 PSI. Balch-type anaerobic culture tubes with butyl stoppers were employed to maintain anaerobicity throughout.

### Cultivation of *Cupriavidus basilensis* 4G11

*Cupriavidus basilensis* 4G11 was retrieved from a previously isolated culture from the Oak Ridge Field Research Center Site by Ray et al. (2015). To recover the strain from frozen glycerol stocks, it was initially plated on R2A medium agar plates and incubated overnight until colonies formed. A single colony was used to inoculate a starter culture (5 mL) of DM9 (Supplementary Table 1) medium containing 25 mM sodium acetate at pH 7.2 incubated at 30 °C. After overnight incubation, this culture was centrifuged and washed twice with sterile water to remove residual media before a final resuspension in PBS. Washed cells were used to inoculate experimental conditions at 1% v/v. Optical density of *C. basilensis* cultures was measured at a wavelength of 600 nm using a spectrophotometer (Genesys 30 visible spectrophotometer, thermo fisher scientific part number 76308-728).

### PHB Cell Biomass Preparation and HPLC Analysis

Bacterial culture samples were centrifuged and dried before digestion in 99.99% sulfuric acid (Sigma) for 30 min at 90°C; vortexing briefly at 10 min intervals, according to the protocol of Tyo et al. with variations mentioned herein (Tyo et al., 2006). The acid-digested samples were then allowed to cool to room temperature for 30 min prior to filtering the samples through a 0.2 μm PVDF syringe filter (Pall part number 4406). PHB standards were prepared by quantitatively dissolving PHB powder (Sigma–Aldrich part number 363502-10G) in chloroform and aliquoting requisite amounts, after which the chloroform was allowed to evaporate overnight. The dried PHB standards were then processed alongside the dried cell biomass samples and analyzed as described below.

Poly(3-hydroxybutyrate) content was measured in processed samples using a Shimadzu Prominence HPLC system equipped with a Reactive Index Detector. Processed samples were eluted and separated using an Aminex HPX-87H column (BioRad part number 1250140) equipped with a micro-guard cation H guard column (BioRad part number 1250129) heated to 40°C and using 5 mM H_2_SO_4_ as the mobile phase flowing at 0.6 mL/min. PHB was enumerated as crotonic acid monomers (products of the concentrated acid digestion protocol mentioned above) at 206 nm. Pure crotonic acid (Sigma Aldrich part number 113018), diluted in 5 mM H2SO4, was also analyzed to verify accurate crotonic acid retention time.

### Dilution and Aeration of Media

The spent and acetate-containing *S. ovata* medium was depressurized and exposed to air prior to *C. basilensis* inoculation. The bottle was loosely covered with a sterile cap and shaken gently for 12 h to aerate. If necessary, the medium was diluted with sterile water to a final acetate concentration of 25 mM. Cultures of the aerated medium (400 mL) were inoculated at 1% v/v with washed *C. basilensis* culture.

### Calculation of Carbon Yield and PHB Productivity

Poly(3-hydroxybutyrate) productivity was calculated by considering the peak rate of PHB synthesis during the 32-h growth period. The net mass of PHB was divided by the window of time within which it was produced to give units of g PHB L^–1^ hr^–1^. Carbon yield from acetate to PHB was derived from the ratio of acetate-carbon consumed to PHB-carbon produced within the window of peak PHB production rate. The units for this metric are reported as % and reflect the fraction of carbon atoms from acetate that were used for PHB synthesis.

### SEM Imaging

Culture aliquots were taken from the *S. ovata*/*C. basilensis* mixed culture at specific time points. These aliquots were supplemented with glutaraldehyde at 2.5% (v/v) and kept at room temperature overnight. The samples were washed and consecutively dehydrated with 10, 25, 50, 75, 90, and 100% ethanol each for 10 min. 50 μL of dehydrated samples were dropcasted on a 1 × 1 cm silicon substrate, allowed to dry and imaged with a Quanta 3D field-emission gun scanning electron microscopy (SEM) (FEI) operated at 10 kV accelerating voltage after gold sputtering.

### Modeling of *S. ovata* Growth and Acetate Production With Periodic Replenishment of H_2_/CO_2_

#### Liquid Phase Mass/Mole Balances

Growth of cells can be modeled according to the standard design equation for a well-mixed batch bioreactor (Harvey and Blanch, 1997).

(1) $$\frac{d\left(c_{\text{X}}V_{\text{L}}\right)}{dt}=\mu V_{\text{L}}c_{\text{X}}$$

where, *c*_X_ is the concentration of cells, *V*_L_ is the liquid volume of the reactor, and μ is the specific growth rate (hr^–1^). We assume that acetate is a purely growth-associated product, resulting in:

(2) $$\frac{d\left(c_{\text{Ac}}V_{\text{L}}\right)}{dt}=\alpha \mu V_{\text{L}}c_{\text{X}}$$

where, α is the molar ratio of growth-associated product formed to cells produced. Solubilized (in the liquid phase) H_2_ and CO_2_ are consumed during the production of biomass and acetate, and mass transfer from the gas phase occurs simultaneously. For H_2_ in the liquid phase, the resulting balance is:

(3) d(cH2VL)dt=VL(-1Y′X/H2μcX-1Y′Ac/H2αμcX)+VLkLa(csat,H2-cH2)

where, Y′X/H2 is the molar biomass yield on H_2_, Y′Ac/H2 is the acetate yield on H_2_, *k*_L_*a* is the gas-liquid mass transfer rate (hr^–1^) and *c*_sat,H_2__ is the saturation concentration of H_2_. The saturation concentration is given, to a reasonable approximation, by:

(4) $$c_{\mathrm{sat},{\text{H}}_{2}}=H_{{\text{H}}_{2}}p_{{\text{H}}_{2}}$$

where, *H*_H_2__ is Henry’s constant for H_2_ in water and *p*_H_2__ is the partial pressure of H_2_ in the gas phase.

Equations (3, 4) are also valid for CO_2_:

(5) d(cCO2VL)dt=VL(-1Y′X/CO2μcX-1Y′Ac/CO2αμcX)+VLkLa(csat,CO2-cCO2)

(6) $$c_{\mathrm{sat},{\text{CO}}_{2}}=H_{{\text{CO}}_{2}}p_{{\text{CO}}_{2}}$$

#### Gas Phase Mole Balances

In the gas phase, H_2_ and CO_2_ are consumed by mass transfer into the liquid phase according to:

(7) $$\frac{d\left(p_{{\text{H}}_{2}}V_{\text{G}}\right)}{dt}=-V_{\text{L}}k_{\text{L}}a\left(c_{\mathrm{sat},{\text{H}}_{2}}-c_{{\text{H}}_{2}}\right)RT$$

and

(8) $$\frac{d\left(p_{{\text{CO}}_{2}}V_{\text{G}}\right)}{dt}=-V_{\text{L}}k_{\text{L}}a\left(c_{\mathrm{sat},{\text{CO}}_{2}}-c_{{\text{CO}}_{2}}\right)RT$$

Note that these equations assume the gas follows ideal behavior.

#### Equation Coupling and Monod Growth Kinetics

We assume that the specific growth rate follows Monod kinetics. In this case, μ can be written as:

(9) $$\mu =\mu_{\text{max}}\left(\frac{c_{{\text{H}}_{2}}}{K_{S,{\text{H}}_{2}}+c_{{\text{H}}_{2}}}\right)\left(\frac{c_{{\text{CO}}_{2}}}{K_{S,{\text{CO}}_{2}}+c_{{\text{CO}}_{2}}}\right)$$

Because both *V*_*L*_ and *V*_*G*_ are constant, the liquid phase mass/mole balances are:

(10) $$\frac{dc_{\text{X}}}{dt}=\mu_{\text{max}}c_{\text{X}}\left(\frac{c_{{\text{H}}_{2}}}{K_{S,{\text{H}}_{2}}+c_{{\text{H}}_{2}}}\right)\left(\frac{c_{{\text{CO}}_{2}}}{K_{S,{\text{CO}}_{2}}+c_{{\text{CO}}_{2}}}\right)$$

(11) $$\frac{dc_{\text{Ac}}}{dt}=\alpha \mu_{\text{max}}c_{\text{X}}\left(\frac{c_{{\text{H}}_{2}}}{K_{S,{\text{H}}_{2}}+c_{{\text{H}}_{2}}}\right)\left(\frac{c_{{\text{CO}}_{2}}}{K_{S,{\text{CO}}_{2}}+c_{{\text{CO}}_{2}}}\right)$$

(12) $$\frac{dc_{{\text{H}}_{2}}}{dt}=\left(-\frac{1}{Y_{X/{\text{H}}_{2}}}\mu c_{\text{X}}-\frac{1}{Y_{\mathrm{Ac}/{\text{H}}_{2}}}\alpha \mu c_{\text{X}}\right)+k_{\text{L}}a\left(c_{\mathrm{sat},{\text{H}}_{2}}-c_{{\text{H}}_{2}}\right)$$

(13) $$\begin{array}{c} \frac{dc_{{\text{CO}}_{2}}}{dt}=\left(-\frac{1}{Y_{X/{\text{CO}}_{2}}}\mu c_{\text{X}}-\frac{1}{Y_{\mathrm{Ac}/{\text{CO}}_{2}}}\alpha \mu c_{\text{X}}\right)\\ +k_{\text{L}}a\left(c_{\mathrm{sat},{\text{CO}}_{2}}-c_{{\text{CO}}_{2}}\right) \end{array}$$

Mole balances in the gas phase result in:

(14) $$\frac{dp_{{\text{H}}_{2}}}{dt}=-\frac{V_{\text{L}}}{V_{\text{G}}}k_{\text{L}}a\left(c_{\mathrm{sat},{\text{H}}_{2}}-c_{{\text{H}}_{2}}\right)RT$$

and

(15) $$\frac{dp_{{\text{CO}}_{2}}}{dt}=-\frac{V_{\text{L}}}{V_{\text{G}}}k_{\text{L}}a\left(c_{\mathrm{sat},{\text{CO}}_{2}}-c_{{\text{CO}}_{2}}\right)RT$$

#### Periodic Gas Phase Replenishment

To describe the periodic (24-h) replacement of the gas phase, we generalize the initial conditions for H_2_ and CO_2_ pressure as:

$$p_{{\text{H}}_{2}}\left(t=24n\right)=p_{{\text{H}}_{2},0}$$

(16) $$p_{{\text{CO}}_{2}}\left(t=24n\right)=p_{{\text{CO}}_{2},0}$$

where, *n* ∈ *ℕ* and *t* has units of hrs.

### Modeling of *C. basiliensis* Growth and PHB Accumulation Fed by *S. ovata* Spent Medium

#### Liquid Phase Mole Balances

We again use the standard bioreactor design equation:

(17) $$\frac{d\left(c_{\text{X}}V_{\text{L}}\right)}{dt}=\mu_{\text{X}}V_{\text{L}}c_{\text{X}}$$

To describe the accumulation of *C. basiliensis* cells in the liquid volume. Following Mozumder et al. (2014) the accumulation of PHB is given as:

(18) $$\frac{d\left(c_{\text{PHB}}V_{\text{L}}\right)}{dt}=\mu_{\text{PHB}}V_{\text{L}}c_{\text{X}}$$

Acetate is consumed both by biomass and PHB accumulation, resulting in:

(19) $$\frac{d\left(c_{\text{Ac}}V_{\text{L}}\right)}{dt}=-\frac{1}{Y_{X/\mathrm{Ac}}^{\prime }}\mu_{\text{X}}V_{\text{L}}c_{\text{X}}-\frac{1}{Y_{\mathrm{PHB}/\mathrm{Ac}}^{\prime }}\mu_{\text{PHB}}V_{\text{L}}c_{\text{X}}$$

Nitrogen is also consumed by biomass accumulation, described as:

(20) $$\frac{d\left(c_{\text{N}}V_{\text{L}}\right)}{dt}=-\frac{1}{Y_{X/N}^{\prime }}\mu_{\text{X}}V_{\text{L}}c_{\text{X}}$$

As above, the liquid volume is approximately constant throughout the duration of the experiment.

#### Growth and PHB Accumulation Kinetics

We use acetate and nitrogen inhibition-modified Monod (Andrews–Haldane) kinetics to describe the kinetics of cell growth:

(21) $$\mu_{\text{X}}=\mu_{\max,X}\left(\frac{c_{\text{Ac}}}{c_{\text{Ac}}+K_{S,\mathrm{Ac}}+\frac{c_{\text{Ac}}^{2}}{K_{I,\mathrm{Ac}}}}\right)\left(\frac{c_{\text{N}}}{c_{\text{N}}+K_{S,N}+\frac{c_{\text{N}}^{2}}{K_{I,N}}}\right)$$

where, *K*_I,i_ are inhibition constants. We modify Mozumder et al. (2014) to describe PHB accumulation:

(22) $$\begin{array}{c} \mu_{\text{PHB}}=\mu_{\max,\mathrm{PHB}}\left(\frac{c_{\text{Ac}}}{c_{\text{Ac}}+K_{S,\mathrm{Ac}}+\frac{c_{\text{Ac}}^{2}}{K_{I,\mathrm{Ac}}}}\right)\left[1-{\left(\frac{f_{\text{PHB}}}{f_{\mathrm{PHB},\max}}\right)}^{\beta }\right]\\ \left(\frac{K_{S,N}}{c_{\text{N}}+K_{S,N}}\right) \end{array}$$

where, *f*_PHB,max_ is the maximum PHB-to-biomass ratio. In this model we neglect O_2_ because O_2_ was fully saturated throughout the duration of the experiment. We also neglect the potential PHB consumption due to cell growth once the acetate source is exhausted because PHB is the intended product, so careful process design, e.g., whereby cells are harvested before PHB consumption occurs, can avoid this parasitic impact on PHB accumulation. All model parameters are compiled in Supplementary Tables 2, 3.

## Results

### Optimization of Base Medium for Bioprocess

**FIGURE 2:**
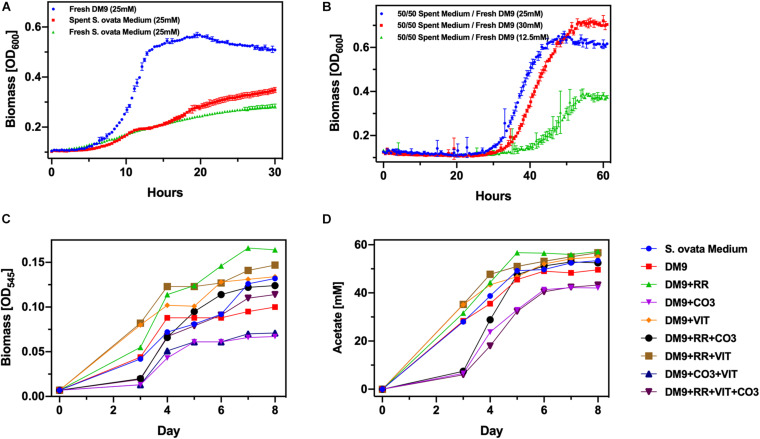
Optimization of media compatibility **(A)** Biomass yield of *Cupriavidus basilensis* in DM9, and in spent and fresh *Sporomusa ovata* medium. **(B)** Biomass yield of *C. basilensis* in spent *S. ovata* medium diluted with fresh DM9 to a range of acetate concentrations. **(C)** Biomass and **(D)** acetate yield of *S. ovata* in DM9 with additional components (RR, reducing reagent; VIT, vitamins; CO_3_, sodium bicarbonate) compared to those in *S. ovata* medium.

We firstly cultured *S. ovata* autotrophically in balch-type 25 mL culture tubes with an 80/20% H_2_/CO_2_ headspace. In these conditions acetate generation rate amounts to 10.4 mmol L^–1^day^–^, a rate modestly higher than previous reports (Blanchet et al., 2015). We selected 25 mM acetate as the feedstock concentration for *C. basilensis*. This titer of acetate was achieved within the first 48–72 h of the autotrophic *S. ovata* culture. However, the *S. ovata* culture could reach a concentration of 50–60 mM acetate in 5–7 days without adjusting for pH which decreases due to acetate accumulation. The *S. ovata* cultures were stopped at 25 mM acetate and aerated which rendered the *S. ovata* inactive. *C. basilensis* was then inoculated in the spent *S. ovata* cultures (Figure 2A). The increase in biomass as detected by OD_600_ was used to monitor the ability of *C. basilensis* to grow and use the CO_2_-derived acetate. The baseline OD resulting from the presence of *S. ovata* biomass in the spent medium was deducted. As compared to *C. basilensis* grown in fresh DM9, which is the recommended *C. basilensis* culture medium, with synthetic acetate the biomass yield in the spent *S. ovata* medium was only 50% within the timeframe of the experiment. We employed a second control with cultured *C. basilensis* in fresh *S. ovata* medium (no prior *S. ovata*) with synthetic acetate. This culture achieved the same biomass yield as the one in the spent *S. ovata* medium indicating that acetate is not incompatible with *C. basilensis* but rather that a component in the *S. ovata* medium inhibits *C. basilensis* growth. In a second medium optimization experiment, we again cultured *S. ovata* autotrophically until the acetate concentration reached 25 mM. The spent *S. ovata* medium was diluted twofold with fresh DM9 containing synthetic acetate at 35, 25, and 0 mM. Thus the mixed media which were inoculated with *C. basilensis* contained total acetate concentrations of 30, 25, and 12.5 mM, respectively (Figure 2B). The biomass yield in the 25 mM culture equaled that of *C. basilensis* in fresh DM9 in the prior experiments. The biomass yields in the 30 and 12.5 mM were congruent with the available acetate. While adding fresh DM9 clearly enhanced *C. basilensis* growth, the cultures containing spent *S. ovata* medium had a 20 h longer lag phase. The peak optical densities were only reached in twice the length of time. Furthermore, it is burdensome to formulate two sets of media. Therefore, we attempted to grow *S. ovata* directly in deoxygenated DM9. We found that a 10% (v/v) inoculum is necessary when culturing *S. ovata* in DM9 whereas normally only a 5% (v/v) would be required. Next, we employed culturing controls in which we added the recommended *S. ovata* medium components to a DM9 base including reducing reagent, vitamins and carbonate (Figures 2C,D). Favorably *S. ovata* not only grows well in DM9 but also generates a similar amount of acetate as in the recommended *S. ovata* medium. Significant decreases in growth and acetate generation are only detected in the carbonate containing DM9 which could be a result of the increased osmotic pressure of the saline medium (Wood, 2015).

### Process Integration for PHB Production

**FIGURE 3:**
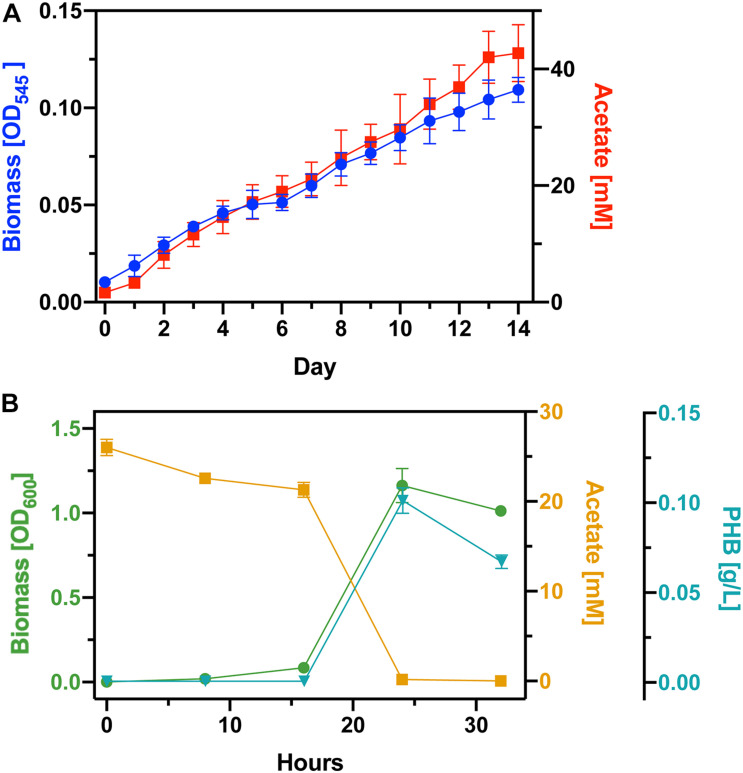
Full bioprocess integration. **(A)** Cell growth and acetate generation by *S. ovata* in DM9 **(B)** Biomass yield, acetate consumption, and PHB accumulation by *C. basilensis* acetate rich spent DM9.

Based on the results from the previous experiments we concluded to use DM9 as the base medium for our bioprocess. Before inoculating with *S. ovata*, we deoxygenated the DM9 medium. To gauge PHB productivity, we scaled up our process from 25 mL tubes to 1L balch-type bottles each with 270 mL of culture medium (Figure 3A). There was a significant scale-up associated loss in acetate productivity with the cultures reaching 25 mM only after 8 days. The *S. ovata* cultures were grown for 14 days and the acetate concentration reached 42 mM. We proceeded to culture *C. basilensis* in the aerated spent DM9 which was diluted to 25 mM (Figure 3B). As expected, the acetate concentration decreases as biomass accumulates. The biomass yield achieved in this experiment was decidedly greater than that obtained in fresh DM9 in Figure 2A. Additionally, there is no protracted lag phase with the use of 100% DM9 as the base medium. Next, we determined that PHB correlates well with biomass production and we calculated a PHB generation rate of 12.6 mg L^–1^ hour^–1^ with an overall 11.06% carbon yield from acetate. After 24 h there is a decrease in the PHB concentration which correlates with the complete consumption of acetate. This could be due to the cells depleting PHB reserves to acquire carbon and energy.

### Qualitative Cell Population Analysis

**FIGURE 4:**
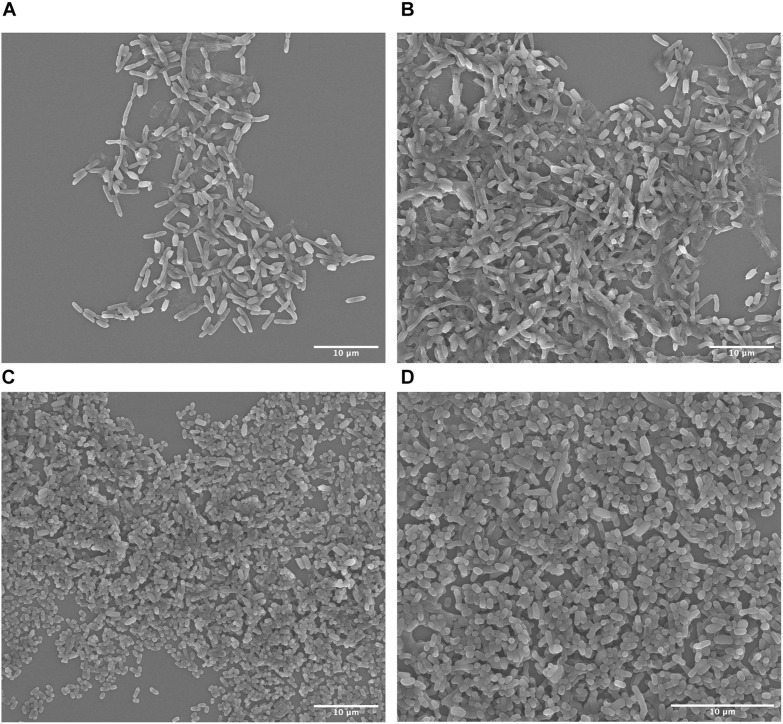
Scanning electron micrographs of bioprocess. **(A,B)** 0 h time point post *Cupriavidus basilensis* inoculation in *S. ovata* containing spent DM9. **(D,C)** 24 h time point after *C. basilensis* exponential growth.

Scanning electron microscopy has been established as a standard method to elucidate bacterial cell morphology. *S. ovata* is rod-shaped circa 4 μm long and 1 μm wide whereas *C. basilensis* is spherical to oval shaped and circa 2 μm in diameter. These differences in morphology allowed us to qualitatively determine the changing cell populations and establish that *C. basilensis* can grow in spent medium containing inactive *S. ovata*. We fixed culture samples at the 0 h time point just after inoculating the spent DM9 with *C. basilensis*. As *S. ovata* had achieved approximately 0.12 OD_545_, we expected to mostly detect *S. ovata*. On the SEM micrographs rod-shaped *S. ovata* is abundantly visible with very few *C. basilensis* cells (Figures 4A,B). Additionally, we fixed culture samples at the 24 h time point after the exponential growth of *C. basilensis*. At this stage, *C. basilensis* cells clearly dominate the landscape (Figures 4C,D). *C. basilensis* closely co-exist with inactive *S. ovata* cells, evidently pointing to the fact that *S. ovata* cells are at least innocuous to *C. basilensis* and underscoring the ability to use spent *S. ovata* medium directly with minimal processing.

### Bioprocess Modeling

**FIGURE 5:**
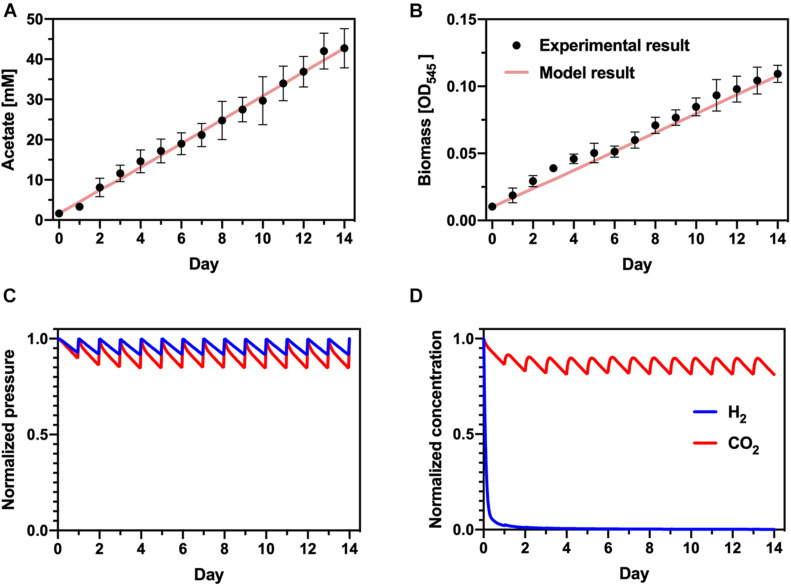
Acetogenesis model. Comparison of experimental data and modeling results for **(A)** acetate and **(B)** biomass accumulation. **(C)** Calculated normalized H_2_ and CO_2_ partial pressures in the headspace of the bioreactor. **(D)** Calculated normalized dissolved concentrations of H_2_ and CO_2_ in the liquid phase of the acetogenesis bioreactor.

To determine process parameters that limit the production rate of PHB, we developed simple models of each step in the bioprocess: H_2_-driven acetate production with *S. ovata* and acetate-driven PHB production with *C. basiliensis*. Experimental data of acetogenesis with *S. ovata* was fit well by adjusting the ratio of carbon diverted to acetate or biomass (α) and the gas-liquid mass transfer coefficient (k_L_a) (Figures 5A,B). Because CO_2_ solubility (∼33 mM/bar) is much greater than H_2_ solubility (∼0.78 mM/bar), this indicates that H_2_ transfer from the gas phase to the liquid phase limits the acetate production rate. Modeled partial pressures in the gas phase (Figure 5C) and concentrations in the liquid phase (Figure 5D) support this conclusion: daily replenishment of the headspace maintains partial pressures of both substrate gasses at > 85% of their initial value. Liquid-phase CO_2_ is similarly maintained at > ∼80% of its initial value. However, H_2_ is nearly completely consumed in the liquid phase within ∼24 h and the concentration does not recover as the gas headspace is replenished, indicating that microbial growth and acetate production consumes H_2_ nearly immediately as it is transferred into the liquid phase.

**FIGURE 6:**
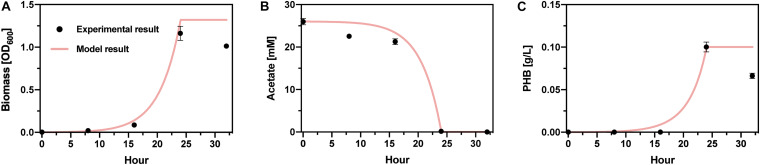
*Cupriavidus basilensis* bioreactor model. Comparison of experimental and model results for **(A)** biomass yield, **(B)** acetate consumption, and **(C)** PHB accumulation.

Experimental PHB production is similarly fit well to a simple Monod-like model of biomass growth and PHB accumulation (Figures 6A–C). Because specific biomass and PHB accumulation rates are determinant of the PHB production and acetate consumption rates, and acetate consumption is complete within ∼24 h, this portion of the integrated bioprocess does not limit overall productivity. Hence, H_2_ gas-liquid mass transfer is the central bottleneck in the full process.

## Discussion

Previous works have described the production of acetate from CO_2_ by *S. ovata* and the production of PHB by closely related *Cupriavidus* strains from acetate (Kedia et al., 2014; Aryal et al., 2017). The traditional manufacturing approach to linking two such processes would be to purify the acetate intermediate between synthesis steps. This process is costly in the terrestrial industry and that cost would be prohibitively magnified in the context of space travel. Here, we have demonstrated the viability of serial biocatalyst culturing in a single media batch without processing of intermediate compounds.

Toward this goal, we sought to formulate a minimal medium that would support growth of both bacterial strains. It was determined that DM9 medium was suited for this task as it was already the preferred medium for *C. basilensis* growth and we observed only a minimal reduction of productivity for *S. ovata* when grown in DM9 compared to the traditional media for this strain. DM9 is an excellent media for space applications due to its extremely simple composition. The media as used here is primarily composed of phosphate buffer which serves to maintain a stable pH throughout bacterial growth. While pH regulation through buffering is common practice for bench-scale, batch reactors, it is highly uncommon and inefficient for large scale industrial settings. The alternative is continuous pH monitoring of the reaction and automated adjustment as needed with concentrated acids and/or bases (Jeevarajan et al., 2002). Therefore, our process in an industrial or space setting would require even fewer components.

Another component of the DM9 medium is the ammonium salt used as a nitrogen source for both strains. Fixed nitrogen could potentially be sourced from the Martian atmosphere as a nitrogen source is likely still required for *S. ovata* growth (Liu et al., 2017). However, it could also likely be favorable to reduce the nitrogen available to *C. basilensis*. It has been shown elsewhere that nutrient starvation, and especially nitrogen, is key to triggering the PHB accumulation mechanisms in *C. basilensis* strains (Mozumder et al., 2014). Therefore, sequestering a high density of active, PHB-poor cells into a nitrogen limited environment might improve the productivity and carbon-flux from acetate to PHB substantially.

We produced bio-acetate to a final concentration of ∼42 mM over the course of 14 days. By modeling this process, we can predict that the rate limiting step of acetogenesis is the gas to liquid transfer rate of H_2_. As this experiment was only performed in shaken balch-type cell cultures bottles with pressurized headspace, there is significant opportunity for operational optimization to increase the gas-to-liquid transfer of H_2_ from the headspace and thus the k_L_a value representing this process. Such improvements may include agitation of the gas-liquid interface by stirring or bubbling. If the k_L_a was increased even modestly to a value of 10, the time required to produce the equivalent mass of acetate would decrease by 75% (Supplementary Material section “Discussion”). Productivity in this stage of the process could be at least doubled in a flow-through, chemostat reactor setting, as opposed to batch, in which bio-acetate laden media is removed continually as it is diluted with fresh media. This approach increases efficiency by keeping the active cell number high and decreasing the need for a time-consuming lag phase in cell growth (Supplementary Material section “Discussion”).

Next, the spent medium containing 42 mM acetate was diluted down to a final concentration of 25 mM acetate. The aeration of the medium at this step was done as a precaution against oxygen limiting conditions but optimization at this phase could prove this unnecessary, especially with the nearly two-fold dilution of the medium in presumably oxygenated fresh liquid. This minimal processing is the key operational cost reduction uncovered by this study.

It was demonstrated clearly that acetate is consumed proportionally to the PHB produced as it is presumed to be the only available carbon source for *C. basilensis*. This assumption is further enforced by the degradation of PHB which occurs after the acetate has been exhausted. In a biomanufacturing setting, acetate levels would need to be monitored so that the PHB could be harvested before it was repurposed as a fuel for biomass accumulation.

The productivity of *C. basilensis* in this study of 12.54 mg PHB L^–1^ hr^–1^ falls below demand of 1.55 g PHB L^–1^ hr^–1^ required for 3D printing an extraterrestrial habitat structure of 12 m^3^ within a reasonable time frame (Menezes et al., 2015). In this demonstration experiment, optimization was not undertaken to improve this metric. The maximal PHB production rate of *Cupriavidus* species when grown in optimized conditions has been reported as high as 1.85 g L^–1^ hr^–1^ (Atlić et al., 2011). We hypothesize that, with additional strain and process optimization, productivity values for our media recycling approach should far exceed its present performance (Kedia et al., 2014; Li et al., 2019; Li and Wilkins, 2020).

Removal of the intermediate purification step between acetate production and PHB production offers both decreased system mass requirements and increased operational simplicity. Purification of acetate from aqueous solution is a delicate and energy intensive task most often performed either by expensive column separation or liquid-liquid extraction followed by distillation. These conventional industrial approaches would prove inefficient and inflexible for continued operation in the context of space travel and may increase the system mass of biological PHB production above the threshold mass of simply bringing pre-made PHB. The method presented here allows for nearly “straight-pipe” integration of the bioprocesses and considerably decreases the complexity of this system.

Even with an increased productivity to 0.04 g PHB L^–1^ hr^–1^, the mass of media components required for generation of 1 kg of PHB would exceed that of simply bringing 1 kg of PHB from Earth. It was estimated that with our current media formulation, 12 kg of media components (primarily phosphate buffer) would be required to produce 1 kg PHB in 1 day (Supplementary Table 1). However, without the straight spent media utilization as presented, the process would require either a distillation column or nearly one ton of ethyl acetate for liquid extraction, assuming 1:1 solvent to media extraction. Altogether, our process affords nearly 98% weight savings as compared to a process involving liquid extraction of acetate. An attractive and thematically aligned approach would be subsequent reuse of media batches for further rounds of acetate and PHB production. While this method was not tested here, studies which determine the rate of nutrient depletion of the media so that it may be supplemented or assigned a maximum life cycle could decrease mass requirements for media significantly. An additional benefit of ISRU as it pertains to producing biopolymer for additive manufacturing is that astronauts would be able to alter the material on demand. A PHA with different material properties could be produced instead of relying on further deployments of goods to fulfill a pressing need.

In all, we have presented an approach for serial biomanufacturing with limited intermediate purification and processing steps. This streamlined approach reduces the need for costly materials that would need to be transported into space in order to support a biomanufacturing facility. Further optimizations of each biocatalyst step should be undertaken to maximize the cost-savings benefit of this process for production of PHB from CO_2_. Lastly, there are additional considerations for microbial production of chemicals and materials in space that are currently under investigation including the effect of cosmic radiation on single-cell organisms, the effect of low gravity environments on CO_2_ conversion and *in situ* resource utilization of Martian water for electrochemical processes.

## Data Availability Statement

The raw data supporting the conclusions of this article will be made available by the authors, without undue reservation.

## Author Contributions

SC-B, SF, APA, and PY designed the experiments. SC-B and JMK carried out *S. ovata* cell work. SF and KBS performed *C. basilensis* culturing. AJA designed the models with experimental inputs. SC-B and SF co-wrote the manuscript with inputs from AJA and KBS. All authors discussed the results and revised the manuscript.

## Conflict of Interest

The authors declare that the research was conducted in the absence of any commercial or financial relationships that could be construed as a potential conflict of interest.

## Publisher’s Note

All claims expressed in this article are solely those of the authors and do not necessarily represent those of their affiliated organizations, or those of the publisher, the editors and the reviewers. Any product that may be evaluated in this article, or claim that may be made by its manufacturer, is not guaranteed or endorsed by the publisher.
