# Supplementary material for: Production of PHB From CO2-Derived Acetate With Minimal Processing Assessed for Space Biomanufacturing
Source: Front Microbiol. 2021 Jul 28;12:700010. doi: 10.3389/fmicb.2021.700010 (PMC8355900; doi:10.3389/fmicb.2021.700010)
Supplement: Supplementary file 1 [file Data_Sheet_1.PDF]

Supplementary Table 1: DM9 recipe

Component	Concentration (per 1L)
**NH_4Cl**	**1 g**
**Na_2HPO_4**	**7 g**
**NaH_2PO_4- $2H_2O$**	**3.9 g**
**$CaCl_2$- $2H_2O$**	**1.47mL of 10 g/L**
**$MgSO_4$- $7H_2O$**	**10mL of 0.1M**

Addition of 2g/L sodium acetate is optional

Supplementary Table 2: S. ovata growth and acetate production parameters

Parameter	Value	Unit	Source
Operating conditions			
T	308.15	K	Experiment
P_{tot}	30	psi	Experiment
$\mathrm{OD}_{545}(t = 0\ \mathrm{hr})$	0.01	--	Experiment
$c_{\mathrm{Ac}}\ (t = 0\ \mathrm{hr})$	1.64	mM	Experiment
Reactor geometry			
$V_{\mathrm{L}}/V_{\mathrm{G}}$	0.43	--	Experiment
Biomass growth			
μ_{max}	0.044	hr^{-1}	[1]
$K_{\mathrm{S,H_2}}$	20	µM	[2]
$K_{\mathrm{S,CO_2}}$	20	µM	[2]
Biomass yield			
$Y'_{\mathrm{X/H_2}}$	0.49	mol mol^{-1}	Element balance
$Y'_{\mathrm{Ac/H_2}}$	0.25	mol mol^{-1}	Element balance
$Y'_{\mathrm{X/CO_2}}$	1	mol mol^{-1}	Element balance
$Y'_{\mathrm{Ac/CO_2}}$	0.5	mol mol^{-1}	Element balance
α	31.5	mol mol^{-1}	Fitted
Gas solubility			
$H_{\mathrm{H_2}}$	0.78	mM bar^{-1}	[3]
$H_{\mathrm{CO_2}}$	33	mM bar^{-1}	[3]
Gas/liquid mass transfer			
$k_{\mathrm{L}}a$	0.405	hr^{-1}	Fitted
Other conversions			
Cell molar mass	25	gCDW mol^{-1}	--
Cell density per OD unit	0.33	g L^{-1} OD^{-1}	--

Supplementary Table 3: C. basilensis growth and PHB production parameters

Parameter	Value	Unit	Source
Operating conditions			
T	303.15	K	Experiment
$OD_{600}(t = 0\text{ hr})$	0.001	--	Experiment
$c_{\text{Ac}}(t = 0\text{ hr})$	26	mM	Experiment
$c_{\text{N}}\ (t = 0\text{ hr})$	10.1	mM	Supplementary Note 1
Biomass growth			
$\mu_{\text{max,X}}$	0.37	hr^{-1}	Fitted (Supplementary Note 2)
$K_{\text{S,Ac}}$	5	µM	Supplementary Note 3
$K_{\text{I,Ac}}$	500	mM	Supplementary Note 3
$K_{\text{S,N}}$	2.05	mM	Fitted
$K_{\text{I,N}}$	120.8	mM	[4]
$Y'_{\text{X/Ac}}$	0.936	mol mol^{-1}	Supplementary Note 4
$Y'_{\text{X/N}}$	5.096	mol mol^{-1}	[4]
PHB Accumulation			
$\mu_{\text{max,PHB}}$	0.095	hr^{-1}	Fitted
$Y'_{\text{PHB/Ac}}$	0.157	mol mol^{-1}	Supplementary Note 4
$f_{\text{PHB,max}}$	6.13	mol mol^{-1}	[4]
β	3.85	--	[4]

Supplementary notes

Note 1: Estimating an initial nitrogen concentration

To estimate the initial nitrogen concentration in the diluted, spent *S. ovata* medium, we used the starting concentration of 1 g/L NH_4Cl (~18.5 mM N), accounted for nitrogen consumption by *S. ovata* using the estimate that 0.24 moles of nitrogen are consumed per mole of biomass (using the biomass formula $CH_{1.77}N_{0.24}O_{0.49}$), and divided the result by 1.8 to account for the dilution of spent *S. ovata* medium prior to inoculation with *C. basiliensis*.

Note 2: Estimating $\mu_{\text{max,X}}$

Because no PHB accumulation occurs during the first 16 hrs of *C. basiliensis* growth and neither acetate nor nitrogen concentrations limit growth during this period, we used these initial time points to fit a maximum growth rate for *C. basiliensis* cells, resulting in an estimate of 0.37 hr^{-1}.

Note 3: Half-saturation and inhibition constants for C. basiliensis *growth on acetate*

We could not find literature estimates of the half-saturation and inhibition constants for *C. basiliensis* growth on acetate, although values as low as 1 µM have been reported for diverse microorganisms.[5,6] We used 5 µM as an estimate, although the actual value doesn't impact model results unless it is >1 mM (data not shown). A similar paucity of data exists for inhibition constants for *C. basiliensis*. However, 750 – 800 mM ranges are common for both *E. coli*[7] and in volatile fatty acid-degrading consortia[8], so we used 500 mM as a conservative estimate for this value. The model fit is only marginally affected provided $K_{\text{I,Ac}}$ > ~150 mM.

Note 4: On biomass and PHB yields on acetate

We similarly could not find literature estimates for biomass and PHB yields on acetate for *C. basiliensis*. However, a wealth of growth data exists with glucose as the substrate for the closely related species *C. necator*[9], which reports $Y_{X/S}$ and $Y_{P/S}$ values of 0.52 g cells g^{-1} glucose and 0.3 g PHB g^{-1} glucose, respectively. Noting that 1 glucose yields 2 pyruvates, and 2 acetates yield 1 pyruvate, we approximate the value of 1 glucose molecule as equal to the value of 4 acetate molecules. This results in a biomass yield of 0.39 g cells g^{-1} acetate, or equivalently 23.4 g cells mol^{-1} acetate, equivalent to the experimental yield of *E. coli* biomass on acetate[10], lending validity to our approach. Following the same strategy results in an estimated PHB yield of 0.225 g PHB g^{-1} acetate.

Supplementary figure and discussion on improvements toward bioprocess scalability

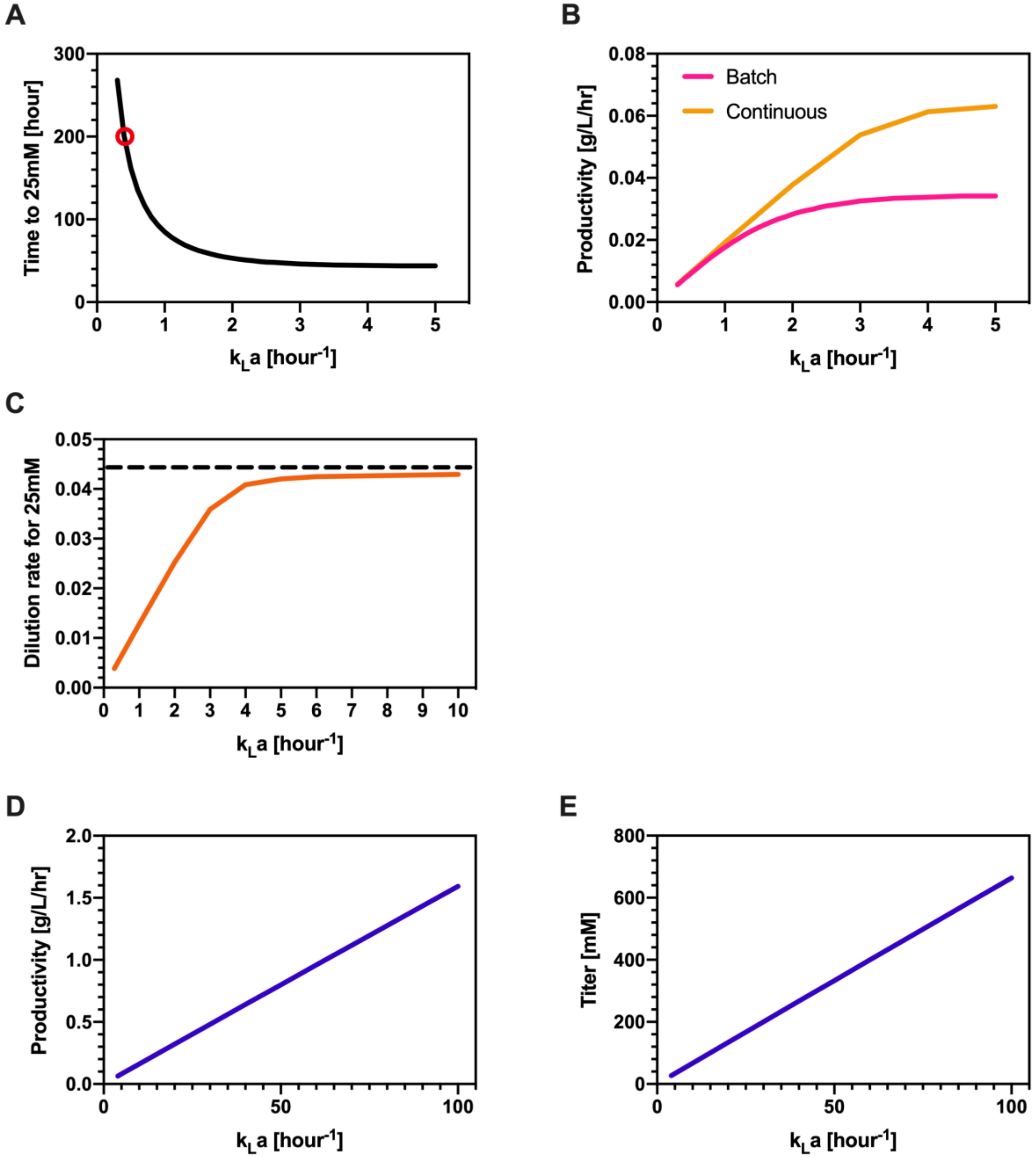

Supplementary Figure 1. Starting with the presented cell culture conditions, we generated a plot **A)** to calculate the time required to reach 25 mM acetate given a particular k_La in a fed batch system. The red circle corresponds to the fitted k_La value from the experimental demonstration. We could decrease the time required to reach 25mM by 150 hours (or ~75%) by increasing the k_La to 10 hr^{-1}. However, the

specific growth rate of *S. ovata* ultimately places an upper limit on the acetate production rate, beyond which further increases in k_La will be of no utility.

Continuous operation enables higher acetate productivity because bacteria can be maintained at relatively high concentrations in the exponential growth phase. Setting the liquid dilution rate to maintain a constant acetate concentration of 25 mM results in a productivity 2-fold higher than the batch case (**B**) at k_La values >~3 hr^{-1}. To maintain 25 mM as k_La increases, the dilution rate must also increase, approaching the maximum specific growth rate of 0.044 hr^{-1} (**C**). However, because the dilution rate cannot exceed the specific growth rate without causing cell washout, productivity in this system is limited to ~0.066 g/L/h. Significantly higher productivities are achievable if the acetate titer is allowed to exceed 25 mM. To illustrate this point, we plot the acetate productivity (**D**) and titer (**E**) as a function of k_La for a fixed dilution rate of 0.04 hr^{-1}. We note that the high acetate concentrations observed at productivities >1 g/L/h could be toxic to *S. ovata* and that, in these cases, active control would be necessary to maintain near-neutral pH in the reactor.

Supplementary Table 4: Weights of process components for different versions of the CO_2 to acetate to PHB bioreaction. Targeting production 1kg PHB assuming production of 1g PHB L^{-1} day^{-1}, translating to 1000L bioreactors in series.

**Parameter**	**Abiotic PHB production**	**Biological production without media recycle**	**Biological production with media recycle (this method)**	**Biological production with media recycle and automated pH adjustment**
Media Components (S. ovata and DM9 media as applicable)	0 kg	14.9 kg	12.0 kg	1 kg
Acetate purification	0 kg	Ethyl acetate (1:1 ratio, 900kg) Distillation column (weight varies)	0 kg	0 kg
Pre-made PHB	1.00 kg	0 kg	0 kg	0 kg
**Approximate Total System Mass**	**1 kg**	**>> 14.9 kg**	**12 kg**	**1 kg**

Potential for further recycle?	No	Yes	Yes	Yes

Supplemental References

1. Shi, X. C., Tremblay, P. L., Wan, L. & Zhang, T. Improved robustness of microbial electrosynthesis by adaptation of a strict anaerobic microbial catalyst to molecular oxygen. *Sci. Total Environ.* **754**, 142440 (2021).
2. Chen, J., Gomez, J. A., Höffner, K., Barton, P. I. & Henson, M. A. Metabolic modeling of synthesis gas fermentation in bubble column reactors. *Biotechnol. Biofuels* **8**, 1–12 (2015).
3. *CRC Handbook of Chemistry and Physics, 84th edition*. (CRC Press, 2004). doi:10.1136/oem.53.7.504
4. Islam Mozumder, M. S., Garcia-Gonzalez, L., Wever, H. De & Volcke, E. I. P. Poly(3-hydroxybutyrate) (PHB) production from CO2: Model development and process optimization. *Biochem. Eng. J.* **98**, 107–116 (2015).
5. Bhattacharya, S. K., Uberoi, V. & Dronamraju, M. M. Interaction between acetate fed sulfate reducers and methanogens. *Water Res.* **30**, 2239–2246 (1996).
6. Chen, F. & Johns, M. R. Substrate inhibition of Chlamydomonas reinhardtii by acetate in heterotrophic culture. *Process Biochem.* **29**, 245–252 (1994).
7. Xiao, Y. *et al.* Kinetic modeling and isotopic investigation of isobutanol fermentation by two engineered escherichia coli strains. *Ind. Eng. Chem. Res.* **51**, 15855–15863 (2012).
8. Vavilin, V. A. & Lokshina, L. Y. Modeling of volatile fatty acids degradation kinetics and evaluation of microorganism activity. *Bioresour. Technol.* **57**, 69–80 (1996).
9. Mozumder, M. S. I., Goormachtigh, L., Garcia-Gonzalez, L., De Wever, H. & Volcke, E. I. P. Modeling pure culture heterotrophic production of polyhydroxybutyrate (PHB). *Bioresour. Technol.* **155**, 272–280 (2014).
10. Andersen, K. B. & Von Meyenburg, K. Are growth rates of Escherichia coli in batch cultures limited by respiration? *J. Bacteriol.* **144**, 114–123 (1980).
